# Mice Expressing A53T/A30P Mutant Alpha-Synuclein in Dopamine Neurons Do Not Display Behavioral Deficits

**DOI:** 10.1523/ENEURO.0170-23.2023

**Published:** 2024-02-06

**Authors:** Cameron Keomanivong, Josephine Schamp, Ervina Tabakovic, Ramasamy Thangavel, Georgina Aldridge, Andrew A. Pieper, Nandakumar S. Narayanan

**Affiliations:** ^1^Carver College of Medicine, University of Iowa, Iowa City 52245, Iowa; ^2^York University, York 68467, Nebraska; ^3^Department of Neurology, University of Iowa, Iowa City 52245, Iowa; ^4^Brain Health Medicines Center, Harrington Discovery Institute, University Hospitals Cleveland Medical Center, Cleveland 44106, Ohio; Department of Psychiatry, Case Western Reserve University, Cleveland 44106, Ohio; Geriatric Research Education and Clinical Center (GRECC), Louis Stokes Cleveland VA Medical Center, Cleveland 44106, Ohio; Institute for Transformative Molecular Medicine, School of Medicine, Case Western Reserve University, Cleveland 44106, Ohio; Department of Pathology, School of Medicine, Case Western Reserve University, Cleveland 44106, Ohio; Department of Neuroscience, School of Medicine, Case Western Reserve University, Cleveland 44106, Ohio; Translational Therapeutics Core, Cleveland Alzheimer’s Disease Research Center, Cleveland, Ohio

**Keywords:** animal models, replication, behavior, dopamine, midbrain

## Abstract

Alpha-synuclein has been implicated in neurodegenerative diseases such as Parkinson's disease and dementia with Lewy bodies, with A53T and A30P mutations shown to be disease causing. It has been reported that hemizygous transgenic mice with tyrosine hydroxylase promotor-driven expression of A53T/A30P mutant alpha-synuclein in dopamine neurons provide a useful preclinical model of these conditions by virtue of developing behavioral deficits. Here, we report a lack of replication of this finding. Despite detecting robust overexpression of A53T/A30P mutant alpha-synuclein in dopamine neurons, we did not observe decreased tyrosine hydroxylase immunofluorescence or behavioral deficits in these mice. Our results demonstrate that preclinical models of synucleinopathy need careful validation in the field.

## Significance Statement

Animal models of synucleinopathy are critical for understanding the basic biology of Parkinson's disease. However, it is well known in the field that many of these animal models are unreliable. This publication attempts to publish negative data on one of these models to encourage others to do the same.

## Introduction

Synucleinopathies such as Parkinson's disease (PD) and dementia with Lewy bodies (DLBs) are poorly understood, difficult to treat, and devastating neurodegenerative disorders. Alpha-synuclein is the major protein found in large intraneuronal inclusions known as Lewy bodies in PD and DLB patients (Spillantini et al., 1998). RT-QuIC assays of alpha-synuclein aggregation are abnormal in PD and DLB patients, and people with mutations or triplications in alpha-synuclein are at increased risk for developing PD or DLB (Singleton et al., 2003). Two common mutations associated with these conditions are A53T/A30P (Conway et al., 1998; Giasson et al., 2022; Li et al., 2002; Kilpeläinen et al., 2019).

To determine how alpha-synuclein contributes to neurodegeneration in these conditions, considerable effort has been devoted in the field to develop mouse models of synucleinopathy. This problem is complex because mice do not live as long as humans (∼2 years vs 60+ years, respectively), do not have elaborated forebrain circuits like humans, and do not express the same forms of alpha-synuclein that are found in humans. In addition, mice without alpha-synuclein have mild deficits (Abeliovich et al., 2000; Kokhan et al., 2012), and mice overexpressing alpha-synuclein through transgenic or viral techniques also have mild behavioral deficits (Chesselet et al., 2012; Decressac et al., 2012; Kim et al., 2017). Notably, mice inoculated with alpha-synuclein pre-formed fibrils (PFFs) in the striatum have reliable behavioral deficits, while mice inoculated with the same PFFs in the cortex do not (Luk et al., 2012; Zhang et al., 2021). Prominent behavioral deficits have been reported in mice with globally expressed A53T mutant alpha-synuclein (Giasson et al., 2022), and mice with global expression of A53T/A30P mutant alpha-synuclein display dopaminergic cell loss and behavioral impairment (Yan et al., 2017; Kilpeläinen et al., 2019).

Expression of A53T/A30P mutant alpha-synuclein in dopaminergic neurons has been reported to specifically induce dopaminergic neuronal cell loss starting at 7–9 months of age, with behavioral deficits manifesting from 13 to 23 months of age (Richfield et al., 2002). This mouse line used a hemizygous allele under selective control of the tyrosine hydroxylase (TH) promoter. Because this hemizygous A53T/A30P mouse line is widely used and publicly available (Jiao et al., 2017; Yan et al., 2017; Tiwari et al., 2022), we attempted to replicate behavioral deficits in this line. However, after driving expression of A53T/A30P mutant alpha-synuclein in dopaminergic neurons and aging mice for 18 months, we did not find deficits in striatal TH, midbrain TH, or behavior in these mice. These data demonstrate that alpha-synuclein mouse models need to be carefully validated in the field to enable investigators to appropriately direct resources toward understanding and finding new ways to understand and treat neurodegenerative disease.

## Materials and Methods

### Mice

Twenty hemizygous [10 female; C57BL/6J-Tg (Th-SNCA*A30P*A53T)39Eric/J] mice were obtained from Jackson Laboratory (hm^2^α-SYN-39), as well as 10 wild-type littermate controls from the same litter (5 female; Richfield et al., 2002). Mice were aged for 18 months prior to final behavioral assessments and histology. One control animal died during the experiment and was excluded. We completed experiments with 29 mice. All experimental procedures were performed in accordance with Protocol #0062039 and approved by the University of Iowa Institutional Animal Care and Use Committee (IACUC).

### Open-field behavior

Mice were placed in a clean open-field container (40 × 40 cm) in a quiet, well-lit room (Meredith and Kang, 2006). Motor and exploratory activity was video recorded and tracked for 10 min using ANY-maze software (Stoelting; Ciobica et al., 2012). Total distance traveled was calculated by ANY-maze software. Odors were removed with 70% alcohol, and the apparatus was allowed to air dry between each trial. Mice were evaluated in this assay at 3, 6, 12, and 15 months.

### Accelerating rotarod

Mice underwent 3 consecutive days of training in which they were placed on a motorized rotarod (Rotamex) with increasing rotation speed from 4 to 40 rpm over a maximum of 10 min (Iancu et al., 2005; Meredith and Kang, 2006). For each training day, mice were provided three opportunities to maintain their perch on the rotarod as rotational speed increased. On the third day, the time it took the mice to fall from the beam was recorded. Mice were evaluated in the rotarod assay at 3, 6, 12, and 16 months. At each age, mice were placed on the apparatus until they fell for a maximum of 10 min. Each test week consisted of 2 d of testing, and mice were given three trials each day.

### Histology

At 18 months when experiments were complete, mice were killed by injections of 100 mg/kg ketamine (Dechra) and transcardially perfused with ice-cold phosphate-buffered saline (PBS). Half the brain was placed in 4% paraformaldehyde (Thermo Fisher), and half the brain was frozen for use in Western blotting. The brains that were removed and post-fixed in paraformaldehyde overnight were immersed in 30% sucrose until the brains sank. Sections (40 µm) were made on a cryostat (Leica) and stored at 4°C. For double immunolabeling, free-floating sections were washed with PBS (Thermo Fisher) and then PBST (0.03% Triton X-100 in PBS; Thermo Fisher), followed by incubation in 2% normal goat serum (NGS; Sigma-Aldrich) for 1 h at room temperature (Zhang et al., 2021; Weber et al., 2023). Next, sections were incubated in primary antibodies of TH (dilution at 1:1,000; AB152; Millipore) and alpha-synuclein (LB509; dilution at 1:100; Invitrogen, catalog #180215) at 4°C overnight. After washing with PBST, sections were incubated with secondary antibodies (goat anti-mouse Alexa 568 and goat anti-rabbit Alexa 488 in 2% NGS) for 1 h at room temperature. After washing again with PBST, and then PBS, sections were mounted onto slides with mounting media containing DAPI (P36962; Invitrogen). Detailed images were captured using a Zeiss confocal microscope (Zeiss). All fluorescent immunohistochemistry images were pseudocolored.

To label TH+ dopaminergic neurons in the striatum and substantia nigra for quantification, sections from WT and hm^2^α-SYN-39 mice were blocked with 2% donkey serum (NDS; Jackson ImmunoResearch Laboratories; catalog #017-000-121) plus 0.3% Triton X-100 in 1× PBS for 1 h at room temperature. After blocking, sections were incubated with primary antibody for overnight at 4°C in the shaker. We used anti-TH primary antibodies (anti-rabbit AB152 at 1:1,000 in 2% NDS; Millipore). On the next day, sections were washed with 1× PBS three times for 5 min and then incubated with secondary antibodies for 1 h (goat anti-rabbit Alexa Fluor 647 at 1:1,000 in 2% NDS; Thermo Fisher). The sections were washed again with 1× PBS for three times for 5 min and then mounted onto Superfrost Plus microslides (Thermo Fisher) and coverslipped with ProLong Diamond antifade mounting medium (Invitrogen). Prior to imaging, the slides were allowed to cure for 48 h in the dark at room temperature and then stored at 4°C in the dark. The slides were scanned using an Olympus slide scanning microscope (Olympus VS120; Olympus) and captured with a 20× objective lens using VS-ASW-S6 imaging software (Olympus). The same fluorescence intensity and exposure time settings were used to capture images for quantification across samples.

To quantify TH fluorescence staining intensity levels in the striatum and substantia nigra, we quantified the mean gray value of the region of interest (ROI) outline for either striatum or substantia nigra. TH immunofluorescence staining intensities for all measured sections were counted for each mouse sample. Outlines of the anterior–posterior coordinate level of striatum (0.22–0.86 mm from bregma) and substantia nigra (−2.8 to −3.3 mm from bregma) regions of interest were based on prior literature and the mouse brain atlas (Paxinos and Franklin, 2013). Data from two hm^2^α-SYN-39 mice were not recoverable for TH quantification.

### Western blotting

**Figure 1.**
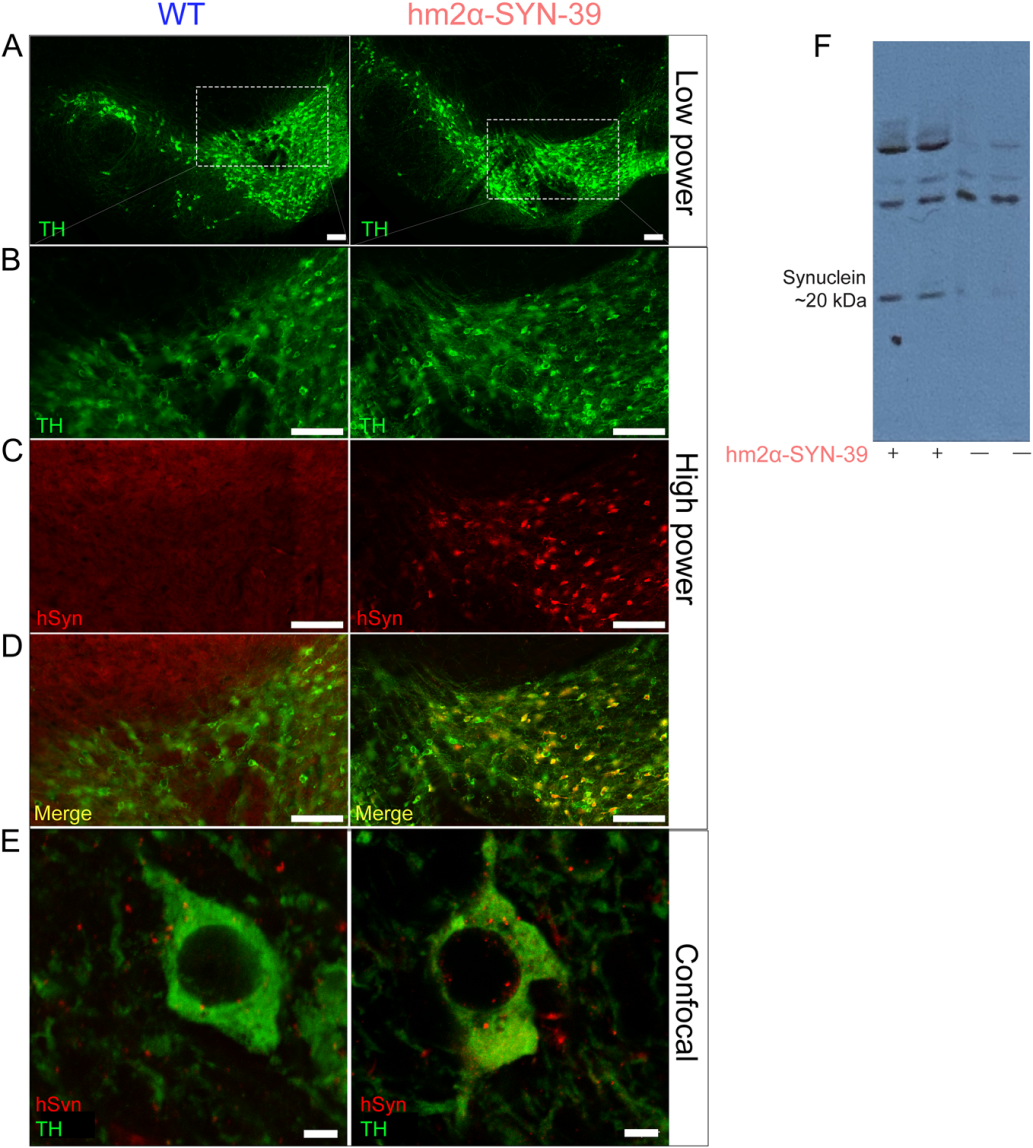
hm^2^α-SYN-39 transgenic mouse overexpress human synuclein. Slide scanning images (20× objective) of a coronal midbrain section through the substantia nigra stained for TH in green in hm^2^α-SYN-39 transgenic mice and wild-type (WT) mice at (***A***) low power (20× objective). ***B***, Subsections from ***A*** outlined in white stained for TH in green. ***C***, Same sections as in ***B*** stained for human synuclein in red, and (***D***) merged images with co-stained neurons in yellow. ***E***, Confocal images (40× objective, 8× digital zoom) of synuclein expression in dopaminergic neurons of the substantia nigra from a wild-type mouse and an hm^2^α-SYN-39 transgenic mouse. Scale bars: ***A–D***, 125 μm; ***E***, 5 μm. ***F***, Western blots from two hm^2^α-SYN-39 transgenic mice and two WT littermate controls.

Protein concentrations per brain sample were determined utilizing a Pierce BCA protein assay kit (Hu et al., 2022; Thermo Fisher). A total of 50 µg of protein were loaded into each well and resolved by gel electrophoresis. Separated proteins were transferred from the gel to a nitrocellulose membrane, which was verified via Ponceau S staining (Martin et al., 2006). Membranes were blocked in TBST (0.1% Tween 20 in TBS) with 5% BSA for 2 h and then incubated with alpha-synuclein monoclonal primary antibody (LB509; Abcam; 1:500) overnight at 4°C and then stained with a goat-anti-mouse secondary antibody (ab205719; Abcam; 1:20,000). After washing with TBST, membranes were incubated with secondary antibody for 2 h at room temperature. Membranes were washed again and then used for enhanced chemiluminescence utilizing the Bio-Rad Clarity Western ECL Substrate (Bio-Rad; Fig. 1*E*).

### Statistics

Analyses were performed using R Statistical Software (v4.1.3; R Core Team 2022).

We tested our hypothesis that alpha-synuclein overexpression causes a loss of TH and behavioral deficits using linear models in R. Outcome variables included rotarod performance or open-field distance traveled, and predictor variables included time and genotype (hm^2^α-SYN-39 or wild-type). We considered main-effect *p* values <0.05 as statistically significant.

## Results

We tested the hypothesis that overexpression of alpha-synuclein in dopamine neurons would lead to neuronal loss and behavioral deficits by comparing to nine littermate wild-type mice to 20 mice expressing A53T/A30P mutant alpha-synuclein under the TH promoter (hm^2^α-SYN-39 mice). Overexpression of mutant alpha-synuclein was verified by immunohistochemistry, showing colocalization of alpha-synuclein with TH+ cells in the substantia nigra (Fig. 1*A–D*). Qualitative, blinded examination by two investigators confirmed increased expression of mutant human alpha-synuclein in the soma of TH+ neurons in the hm^2^α-SYN-39 model compared with wild-type controls by confocal microscopy (Fig. 1*E*). Western blotting also confirmed that wild-type littermate mice did not express human alpha-synuclein, whereas there was expression of mutant alpha-synuclein in transgenic mice (Fig. 1*F*).

**Figure 2.**
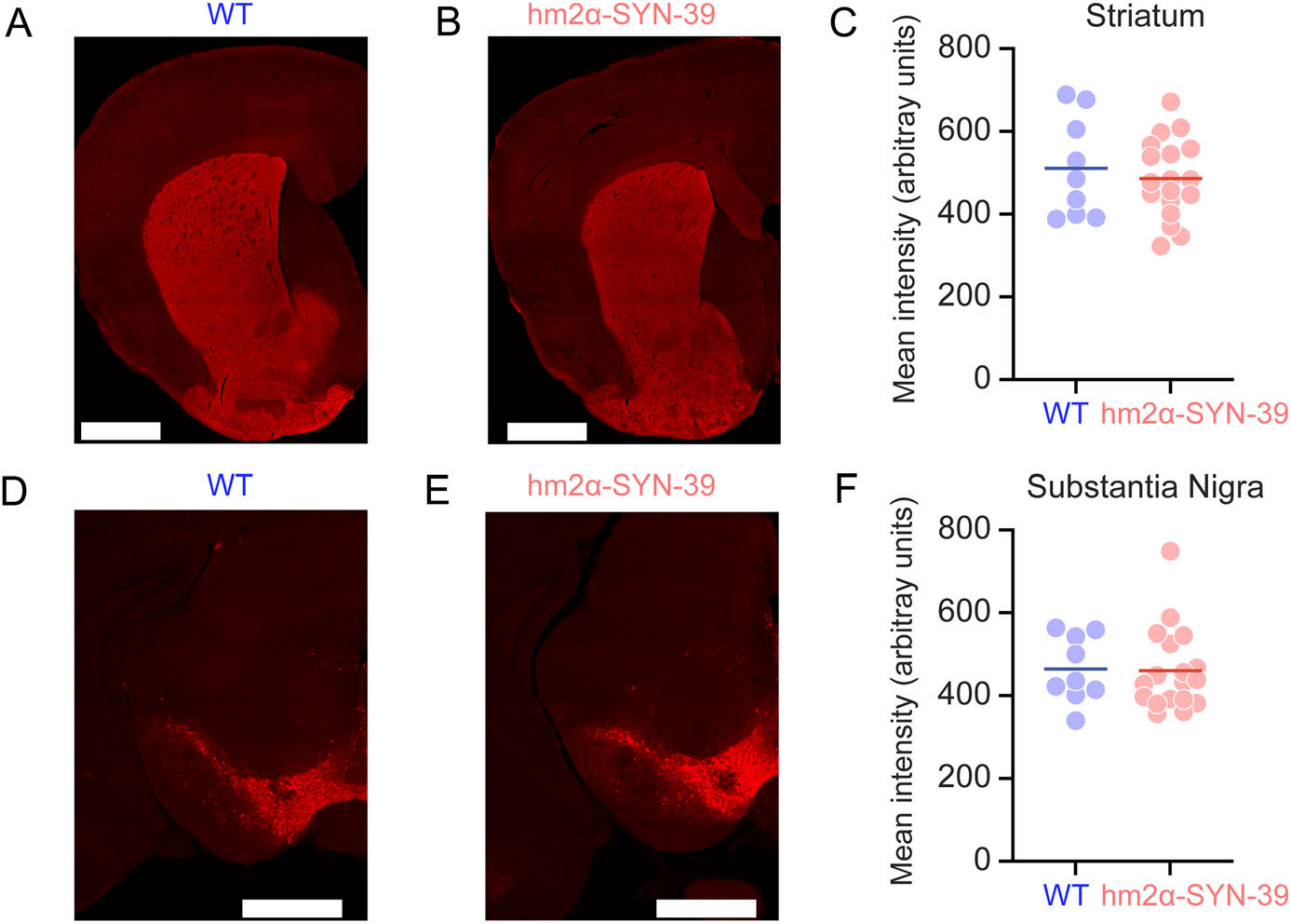
Quantification of TH immunofluorescence in the striatum and substantia nigra is similar in hm^2^α-SYN-39 and wild-type mice. TH staining (red) from the striatum of (***A***) wild-type (WT) and (***B***) hm^2^α-SYN-39 mice. ***C***, Quantification of TH intensity revealed no consistent differences between genotypes. Similarly, TH from the midbrain of (***D***) WT and (***E***) hm^2^α-SYN-39 mouse. ***F***, Quantification of TH levels revealed no consistent differences between genotypes. Scale bars: ***A***, ***B*** and ***D***, ***E***, 1,000 μm. Data from 9 wild-type mice (WT; blue) versus 18 hm^2^α-SYN-39 mice (red).

To determine if there was a change in dopamine neurons in hm^2^α-SYN-39 mice, we quantified the intensity of TH+ immunofluorescence from neurons in substantia nigra and terminals in the striatum. Quantitative measurement of TH+ immunofluorescence revealed no consistent differences between WT mice and hm^2^α-SYN-39 mice in the striatum (Fig. 2*A–C*) or in the midbrain (Fig. 2*D–F*). These data provide evidence that the number of TH+ terminals or cell bodies was not reliably decreased in hm^2^α-SYN-39 mice.

**Figure 3.**
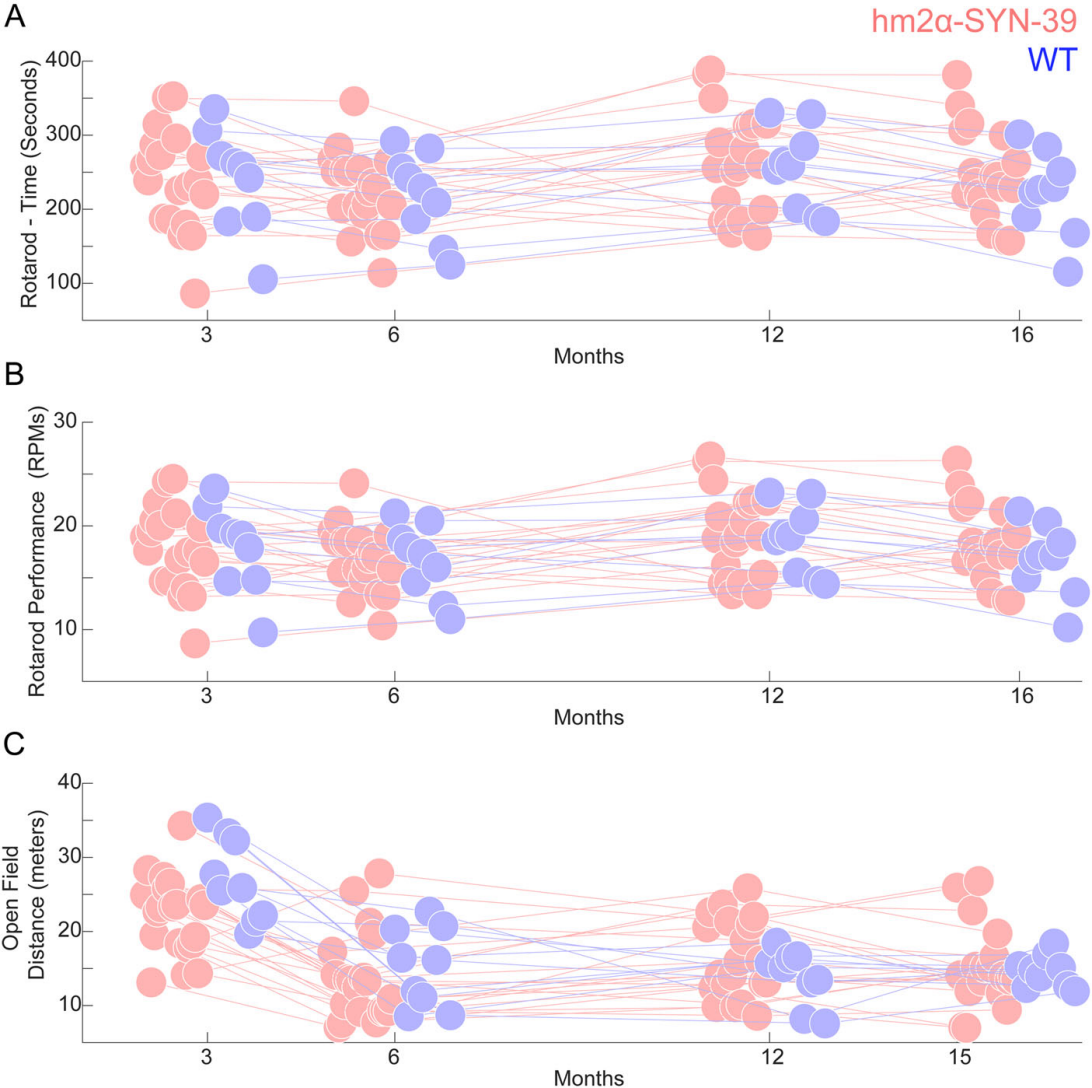
hm^2^α-SYN-39 transgenic mice do not have reliable behavioral deficits. ***A***, Time on the rotarod and (***B***) maximum revolutions per minute (rpm). There were no reliable effects of genotype for 9 wild-type mice (WT; blue) versus 20 hm^2^α-SYN-39 mice (red). ***C***, Open-field distance traveled for WT versus hm^2^α-SYN-39 mice; there was a main effect of time, genotype, and an interaction due to the hm^2^α-SYN-39 mice being less active early and hyperactive late in their lives. Points were jittered for visualization.

Next, we evaluated for behavioral deficits through testing in the accelerating rotarod and open-field tasks in which it was previously reported that these mice displayed deficits at 15 months of age. Performance in the accelerating rotarod task was quantified by measuring the time mice stayed on the rotarod (Fig. 3*A*) and the maximum revolutions per minute attained at the point of falling (Fig. 3*B*). For rotarod performance over 3–16 months, linear-mixed-effects models revealed no main effects of time, synucleinopathy, or higher interactions (Richfield et al., 2002). In the open-field test, we monitored distance traveled (Fig. 3*C*). Though there was a significant effect of time (*F* = 21.8; *p* < 0.00001), an effect of genotype (*F* = 4.4; *p* < 0.04), and an interaction of time with genotype (*F* = 4.7; *p* < 0.03), this was only because hm^2^α-SYN-39 mice were less active initially and then became hyperactive with age (Fig. 3*C*). Importantly, at 15 months of age, there was no difference in performance between transgenic mice and wild-type littermates, in contrast to what was previously reported (Richfield et al., 2002). Thus, the prior findings of behavioral deficits in hm^2^α-SYN-39 mice expressing A53T/A30P mutations cannot be replicated.

## Discussion

We investigated if mice expressing the A53T/A30P mutation in alpha-synuclein in dopaminergic neurons had neurodegeneration and behavioral deficits and found neither to be the case. We found no evidence of TH loss or behavioral deficits in rotarod or open-field behavior, in contrast with the prior report (Richfield et al., 2002). These data suggest that this specific alpha-synuclein model does not reliably produce behavioral deficits, suggesting that mouse alpha-synuclein models must be carefully validated. There were differences in open-field behavior as a function of genotype, but these mice were less active initially. These behavioral findings are not consistent with those noted in published literature, which reported hypoactivity at later ages (Richfield et al., 2002).

This report was published nearly 20 years ago, and there may have been substantial genetic drift or changes. Indeed, genetic drift can affect the penetrance of key genes, their expression or post-translational modification, or the relative toxicity of synuclein (Casellas, 2011). Genetic drift can affect mouse lines despite transgenic maintenance because the trait of interest—behavioral deficits in aged mice—is difficult and time consuming to assess. Lastly, publication bias may contribute to negative findings remaining unpublished (Stanley, 2005).

A recent study further bred this mouse line to be homozygous with additional copies of the A53T/A30P mutation and found early behavioral deficits (Kilpeläinen et al., 2019). Behavioral deficits were apparent at 3 months, whereas we do not find reliable deficits in this study at advanced ages, Richfield et al. (2002) only found deficits after 7–9 months. These data suggest that synuclein gene dosage may contribute to motor deficits in rodent PD models (Singleton et al., 2003; Kim et al., 2017). However, future studies that carefully control synuclein expression will be required to establish the relationship between synuclein gene dosage and deficits.

Interestingly, Kilpeläinen et al. (2019) conducted additional breeding to achieve homozygosity, which likely required several generations. Kilpeläinen et al. (2019)'s homozygous A53T/A30P was distinct from the hemizygous A53T/A30P mutation used in this manuscript. The hemizygous A53T/A30P mutation is readily available from Jackson Laboratories, deposited by Richfield et al. (2002), and is widely used (Jiao et al., 2017; Yan et al., 2017; Tiwari et al., 2022). In contrast, the homozygous A53T/A30P mutation was only used, to our knowledge, in one study (Kilpeläinen et al., 2019). Consequently, the reliability of behavioral phenotypes in the readily available and widely used hemizygous A53T/A30P mutation is of great interest to the PD field.

The mice in homozygous A53T/A30P mouse lines were male (Kilpeläinen et al., 2019). Our study and (Richfield et al., 2002) used both female and male mice. We did not find reliable behavioral deficits in either males or females, although male mice were particularly sensitive to amphetamine in Richfield et al. (2002). Parkinson's disease has a male predominance (Taylor et al., 2007), and future studies will determine how synuclein interacts with sex-dependent factors to contribute to disease pathophysiology.

In addition, there may be distinct environmental differences that affect synuclein pathology, such as variances in the microbiome or other aspects of inflammation (Berardi et al., 2007; Yang et al., 2018; Gorecki et al., 2019). These factors can be challenging to systematically control across contexts. Regardless, we do not find reliable deficits in the A53T/A30P model.

A limitation of our work is that we quantified immunofluorescence for TH and did not directly quantify catecholamines or metabolites. Our data do not directly undermine the synuclein hypothesis; rather, our data suggest that animal models need to be carefully validated, and both positive and negative data need to be published. Our hope is that publication of our negative results will add clarity and direction for the field of synuclein-related neurodegeneration.

## Synthesis

Reviewing Editor: Mikko Airavaara, Helsingin yliopisto - Viikin kampus Decisions are customarily a result of the Reviewing Editor and the peer reviewers coming together and discussing their recommendations until a consensus is reached. When revisions are invited, a fact-based synthesis statement explaining their decision and outlining what is needed to prepare a revision will be listed below. The following reviewer(s) agreed to reveal their identity: NONE.
